# A Pictorial Human Case of “Furious Rabies”

**DOI:** 10.3201/eid3106.250167

**Published:** 2025-06

**Authors:** Charles E. Rupprecht, Alan C. Jackson

**Affiliations:** Auburn University College of Veterinary Medicine, Auburn, Alabama, USA (C.E. Rupprecht); University of Calgary Cumming School of Medicine, Calgary, Alberta, Canada (A.C. Jackson)

**Keywords:** Franz (Ferenc) Paczka, Un Cas Grave ou La Leçon de Médecine, A Pictorial Case of Human “Furious Rabies,” rabies, rabies virus, human furious rabies, iconodiagnosis, viruses

**To the Editor:** We congratulate Perciaccante et al. (*1*) on their article about a Paczka painting showing doctors looking for evidence of a dog bite on a presumed encephalitic rabies patient. However, the story incorrectly references “Émile Roux’s first inoculation of a human with rabies vaccine in July 1885.”

Émile Roux (1853–1933) was a brilliant physician and scientist who began work with Louis Pasteur during 1878. Roux was noted for rabies research and standardized techniques for intracerebral inoculation of animals. In fact, Roux designed the well-known technique of using a flask to inactivate suspended rabies virus–infected rabbit cord through desiccation (an idea Pasteur borrowed). Roux was nominated for the Nobel Prize in 1888 for his research on diphtheria and later became director of the Pasteur Institute (1904–1933). Clinician, researcher, and scholar, Roux was renowned for multiple accomplishments but not his involvement with Joseph Meister’s vaccination.

Roux and Pasteur had many disagreements, including the ethics of human experimentation. After only 5 weeks of animal research, Roux was unconvinced the experimental biologic made from dried spinal cords of infected rabbits was safe for human administration and did not support its use for Meister (*2*). His objection was problematic, because Pasteur, not a physician, could not administer the vaccine. Rather, vaccination proceeded under the supervision of E.F.A. Vulpian and Joseph Grancher. Dr. Grancher, professor, tuberculosis specialist, and Pasteur collaborator, administered the first vaccination, and fortunately, Meister survived (*3*,*4*). That news was shared only after vaccination of Jean-Baptiste Jupille, a 15-year-old shepherd, who was bitten in October and also survived (*5*). “… Roux, who would normally have administered inoculations for Pasteur, was notably absent...” (*6*). Only once evidence had accumulated did Roux relax his stance. Thereafter, he became an adherent of the “Pasteurian method” (*6*). This occurrence demonstrates that success is not without controversy or risk, and experts often disagree, given differing backgrounds, training, philosophy, and ethics.
